# Dynamic Changes of DNA Methylation During Wild Strawberry (*Fragaria nilgerrensis*) Tissue Culture

**DOI:** 10.3389/fpls.2021.765383

**Published:** 2021-11-30

**Authors:** Qiang Cao, Yuxi Feng, Xiongwei Dai, Lin Huang, Jiamin Li, Pang Tao, M. James C. Crabbe, Ticao Zhang, Qin Qiao

**Affiliations:** ^1^School of Agriculture, Yunnan University, Kunming, China; ^2^Horticultural Research Institute, Yunnan Academy of Agricultural Sciences, Kunming, China; ^3^Wolfson College, Oxford University, Oxford, United Kingdom; ^4^Institute of Biomedical and Environmental Science and Technology, School of Life Sciences, University of Bedfordshire, Luton, United Kingdom; ^5^School of Life Sciences, Shanxi University, Taiyuan, China; ^6^College of Chinese Material Medica, Yunnan University of Chinese Medicine, Kunming, China

**Keywords:** *Fragaria nilgerrensis*, tissue culture, somaclonal variations, DNA methylation, gene expression

## Abstract

Tissue culture is an important tool for asexual propagation and genetic transformation of strawberry plants. In plant tissue culture, variation of DNA methylation is a potential source of phenotypic variation in regenerated plants. However, the genome wide dynamic methylation patterns of strawberry tissue culture remain unclear. In this study, we used whole-genome bisulfite sequencing (WGBS) to study genomic DNA methylation changes of a wild strawberry *Fragaria nilgerrensis* at six stages: from explants of shoot tips to outplanting and acclimation. Global methylation levels showed that CG sites exhibited the highest methylation level in all stages with an average of 49.5%, followed by CHG (33.2%) and CHH (12.4%). Although CHH accounted for the lowest proportion of total cytosine methylation, it showed the most obvious methylation change and the most of these changes occurred in the transposable element regions. The overall methylation levels alternately decreased and increased during the entire tissue culture process and the distribution of DNA methylation was non-uniform among different genetic regions. Furthermore, much more differentially methylated regions (DMRs) were detected in dedifferentiation and redifferentiation stages and most of them were transposable elements, suggesting these processes involved activating or silencing of amounts of transposons. The functional enrichment of the DMR-related genes indicated that genes involved in hormone metabolic processes, plant development and the stress response changed methylation throughout the tissue culture process. Finally, the quantitative real-time PCR (qRT-PCR) was conducted to examine the association of methylation and gene expression of a set of different methylated genes. Our findings give deeper insight into the epigenetic regulation of gene expression during the plant tissue cultures process, which will be useful in the efficient control of somaclonal variations and in crop improvement.

## Introduction

The strawberry is one of the most economically important fruits in the world, belonging to the genus *Fragaria* L. (Rosaceae). *Fragaria nilgerrensis* is a widely distributed diploid wild strawberry in southwest China. Its white fruits with a unique peach aroma, as well as strong resistance to drought and cold are valuable characteristics for cultivated strawberry improvement (Noguchi et al., 2002; Guo et al., 2018; Lu et al., 2021). Recently, the genome sequence of *F. nilgerrensis* has been released and it could serve as another ideal model system for genetic studies of strawberry plants, and has great potential in broadening the genetic background of cultivated strawberries (Feng et al., 2020; Qiao et al., 2021).

The plant tissue culture technique is one of the most important tools in modern plant science research, which can be used for rapid asexual reproduction and genetic transformation, as well as an important means to understand the cell totipotency of plants (Ghosh et al., 2021). Under the influence of artificial hormonal environments, plant cells need to reset their genetic and epigenetic programs to adapt to the *in vitro* culture environment, and such molecular dynamic changes can also lead to stable genetic or epigenetic variations in clone progeny, also known as “somatic variation.” These mutations may not be conductive to commercial production from tissue culture, but they are an important source for the development of new varieties with particular characteristics.

Among epigenetic factors, DNA methylation plays an important role in regulating chromatin conformation and gene expression during plant regeneration (Gupta et al., 2006; Ehrlich and Lacey, 2013; Lee and Seo, 2018). It has been reported that alteration of DNA methylation is related to developmental switches occurring during *in vitro* culture, which is determined by several factors including plant growth regulators, genetic backgrounds, and different types of stress (Baránek et al., 2010; Us-Camas et al., 2014; Karim et al., 2016). Recent advances in the field of epigenetics have revealed highly dynamic mechanisms of global and local DNA methylation variations occurring during cell dedifferentiation and redifferentiation processes in callus formation (Horstman et al., 2017; Xia et al., 2017). Few studies have focused on dynamic changes of methylation patterns during the whole process of tissue culture, which is not only crucial for commercial production of disease-free strawberry plants, but also for constructing a genetic transformation system. Understanding the epigenetic landscape and epigenetic mechanisms that modulate gene expression at each stage of tissue culture may be crucial for understanding variant phenotypes. This information can be used in crop improvement programs in a controlled way to generate better agronomic traits based on selection for favorable epigenetic states, creation of novel epialleles and avoided the negative consequences of variation (Tetsu and Akemi, 2013).

Therefore, in the present study, we explored the genome-wide methylation patterns and differences at the CG, CHG and CHH sites of six developmental stages of tissue culture in *F. nilgerrensis.* The differentially methylated regions (DMRs) were detected between each adjacent stage and associated genes with altered methylation were identified. Our results will help to identify the hypervariable regions in the plant genome during the tissue culture process, which should lead to the efficient control of somaclonal variations and their use in crop improvement programs.

## Materials and Methods

### Plant Material and Tissue Culture

Plants of *F. nilgerrensis* were grown in the greenhouse in Yunnan University and conventionally propagated by runners to ensure all the plant materials were from the same clone. Runner tips 1–2 cm long were taken from these plants as explants. Explants were rinsed under running tap water for 30 min and then immersed in 75% alcohol for 20–25 s, followed by 0.1% HgCl_2_ for 7 min. After that, the explants were thoroughly washed (4–5 washings) with sterilized distilled water and then shortened to 3–5 mm long. Finally, they were sampled or transferred to optimized medium for strawberry micropropagation in turn as listed in Table 1. The tissue culture was conducted in an incubation room at 14/10 light/day photoperiod conditions (38 μE m^–2^ s^–1^) at temperatures of 25 ± 2°C for day and 20 ± 2°C for night. In the callus induction stage, dark culture lasting about 10 days was first required. The tissue cultured plantlets were transferred to pots in the greenhouse after proper hardening. The culture medium used for each stage is shown in Table 1. The materials collected from each stage with three biological replicates were shock-frozen in liquid nitrogen immediately and stored at −80°C.

**TABLE 1:** Media formulations at various stages and tissues collected for sequencing.

Stages	Basal medium (pH = 5.8, with 3% sucrose and 7 g/L agar)	Culture time	Materials source
Explants of shoot tips	/	/	Shoot tips
Callus induction	MS + 0.2 mg/L TDZ + 0.6 mg/L 6-BA + 0.15 mg/L 2,4-D + 0.6 mg/L NAA	30 days	Calli
Shoot induction	MS + 1 mg/L 6-BA + 0.1 mg/L NAA	40 days	Leaves
Shoot elongation	MS + 0.1 mg/L NAA + 0.1 mg/L IBA	25 days	Leaves
Rooting	1/2MS + 0.2 mg/L IBA	20 days	Leaves
Outplanting and acclimation	Peat soil:perlite:vermiculite = 3:1:1	30 days	Leaves

### Library Construction and Whole-Genome Bisulfite Sequencing

Genomic DNA was extracted using the Hi-DNAsecure Plant Kit (Qiagen GmbH, Hilden, Germany), according to the manufacturer’s recommendations. Genomic DNA degradation and contamination was monitored on agarose gels. A total of 5.2 ug qualified genomic DNA spiked with 26 ng lambda DNA was fragmented by sonication to 200–300 bp with a Covaris S220 (Covaris, Woburn, MA, United States), followed by end repair and adenylation. Cytosine-methylated barcodes were ligated to sonicated DNA as per the manufacturer’s instructions. Then these DNA fragments were treated twice with bisulfite using the EZ DNA Methylation-Gold™ Kit (Zymo Research, Irvine, CA, United States). The resulting single-strand DNA fragments were amplified by the polymerase chain reaction (PCR) using KAPA HiFi HotStart Uracil + ReadyMix (2X) (Kapa Biosystems, Wilmington, MA, United States). Library concentration was determined with a Qubit 2.0 Flurometer (Life Technologies, CA, United States) and quantitative PCR, and the insert size was assayed on an Agilent Bioanalyzer 2100 system (Agilent, Santa Clara, CA, United States). The prepared library was sequenced on an Illumina Hiseq 2500. Image analysis and base calling were performed with an Illumina CASAVA pipeline, and finally 125 bp/150 bp paired-end reads were generated.

We used FastQC (fastqc_v0.11.5) to perform quality control of the raw reads. Then, adapter sequences and low quality reads were removed through Trimmomatic (Trimmomatic-0.36) software using the following parameters (SLIDINGWINDOW: 4:15; LEADING: 3, TRAILING: 3; ILLUMINACLIP: adapter.fa: 2: 30: 10; MINLEN: 36). The remaining reads that passed all the filtering steps were counted as clean reads and all subsequent analyses were based on these data.

### Reads Mapping to the Reference Genome

We have performed *de novo* genome sequencing of *F. nilgerrensis* (Qiao et al., 2021). Here, we used Bismark software (version 0.16.3) (Karim et al., 2016) to align the bisulfite-treated reads to our sequenced reference genome. The reference genome was firstly transformed into a bisulfite-converted version (C-to-T and G-to-A converted) and then indexed using bowtie2 (Langmead and Salzberg, 2012). Sequence reads were also transformed into fully bisulfite-converted versions (C-to-T and G-to-A converted) before they were aligned to similarly converted versions of the genome in a directional manner. Sequence reads that produced a unique best alignment from the two alignment processes (original top and bottom strand) were then compared to the normal genomic sequence and the methylation state of all cytosine positions in the read was inferred. The same reads that aligned to the same regions of the genome were regarded as duplicated ones. The sequencing depth and coverage were summarized using deduplicated reads.

### Genome-Wide DNA Methylation Distributions Analysis

In order to calculate the methylation level of the sequence, we divided the sequence into multiple bins, with a bin size of 10 kb. The sums of methylated and unmethylated read counts in each window were calculated. Methylation level (ML) for each C site shows the fraction of methylated Cs (mC) and is defined by the following equation: ML = reads (mC)/reads (mC + umC), where umC are the non-methylated Cs.

Calculated ML was further corrected with the bisulfite non-conversion rate according to previous studies (Lister et al., 2013). The calculation was based on the percentage of methylated cytosine in the entire genome, in each chromosome and different regions of the genome, and in three sequence contexts (CG, CHG, and CHH).

### Detection of Differentially Methylated Regions and Their Related Genes

Differentially methylated regions were identified using the DSS package (Wu et al., 2015). The DSS method uses spatial correlation (the level of methylation at sites adjacent to cytosine), the sequencing depth of cytosine sites, and the difference between biological repeats to detect and evaluate DMRs. According to the distribution of DMRs through the genome, we defined the genes related to DMRs as genes whose gene body region (from TSS to TES) or promoter region (upstream 2 kb from the TSS) had an overlap with the DMRs.

### Gene Ontology and Kyoto Encyclopedia of Genes and Genomes Enrichment Analyses of Differentially Methylated Region-Related Genes

Gene Ontology (GO) enrichment analysis of genes related to DMRs was implemented by the GOseq R package (Young et al., 2010), in which gene length bias was corrected. GO terms with corrected p-values less than 0.05 were considered significantly enriched by DMR-related genes. The main feature of KEGG (Kyoto Encyclopedia of Genes and Genomes) is to link genes with various biochemical reactions. We used KOBAS software (Mao et al., 2005) to test the statistical enrichment of DMR-related genes in the KEGG pathways. Similarly corrected pathways with *p*-value < 0.05 were considered to be pathways with significant enrichment of DMR-related genes.

### Quantitative Real-Time PCR Validation of Differentially Methylated Region-Related Genes

We randomly selected 20 differentially methylated region-associated genes (DMGs) with significant changes in methylation level at each stage and verified them by qRT-PCR (Quantitative real-time PCR). Total RNA was extracted from six stage samples using a plant RNA Kit (OMEGA bio-tek, Guangzhou, China). Reverse transcription of total RNA was conducted with the PrimeScript RT kit (Takara, Dalian, China) as per the manufacturer’s protocol. Each complementary DNA sample was assayed on the QuantStudio 7 Flex real time PCR system software (Thermo Fisher Scientific, United States) with TB green Premix Ex Taq II (Tli RNaseH plus) kit. Gene primers for each gene are listed in Supplementary Table S1. The 2^–ΔΔ*Ct*^ method (Livak and Schmittgen, 2001) was employed for normalization of the relative expression of each gene using *FnACTIN* as an internal reference. Each qRT-PCR experiment consisted of three independent biological replicates with two technical replicates for each.

## Results

### Sequencing Samples and General Evaluation of Whole Genome Bisulfite Sequencing

**FIGURE 1:**
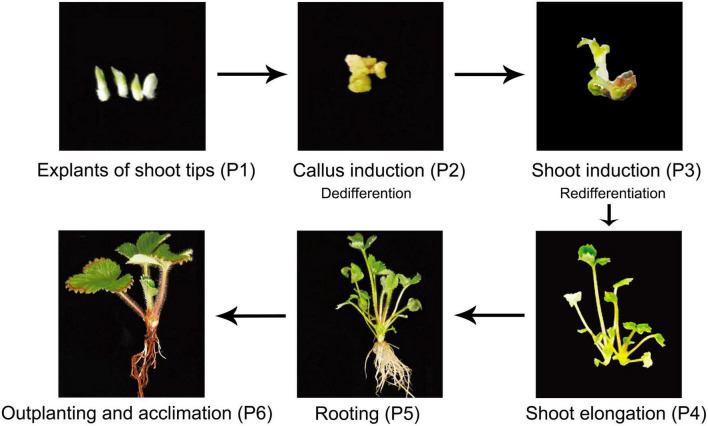
Plant materials used in this study. Plant regeneration of *F. nilgerrensis* from explants of shoot tips to outplanting and acclimation.

Different tissues were collected from six stages of the tissue culture process: shoot tips were sampled from explants stage (P1), calli were sampled from the callus induction stage (P2), leaves were collected from shoot induction (P3), shoot elongation (P4), rooting (P5) and outplanting (P6) stages, respectively (Figure 1 and Table 1). Each sample included three biological replicates for Genome Bisulfite Sequencing. The optimized protocol of strawberry micropropagation used in this study is shown in Table 1, as developed previously in our lab. A total of 18 samples from six stages of the tissue culture process were collected and sequenced. After three types of cytosine methylation were calculated in the three replicates of each stage, we found that the methylation levels of four samples deviated from other corresponding replicates. Therefore, samples P1-3, P3-1, P5-2, P6-3 were eliminated and a total of 14 samples were used for further analysis. All the Pearson correlation coefficients (*R*^2^) among the replicates were >0.95 in three sequences contexts, indicating high reproducibility between stage-specific replicates (Supplementary Figure S1).

A total of 171.44 G raw reads were generated for 14 samples by WGBS. After quality control, 154.38 G clean reads were obtained, with an average of 11 G clean reads per sample; the lowest Q20 and Q30 were 96.72% and 90.25%, respectively (Supplementary Table S2). The unique mapping rate of 14 samples ranged from 60.43–75.33%. The average coverage depth of C sites ranged from 7.2×–13.0× (Supplementary Table S3). At the same time, the BS conversion rate of the sequencing library was >99.294%, indicating that the DNA methylation information on reads was highly reliable.

### DNA Methylation Profiling Varied During Different Stages of Tissue Culture

**FIGURE 2:**
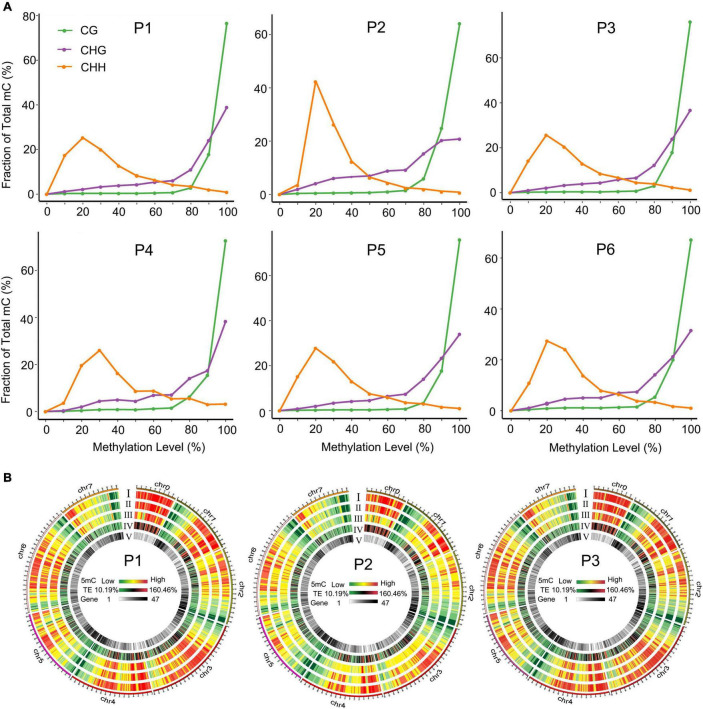
Distribution of methylation levels in different tissue culture stages. **(A)** Distribution of methylation levels in different sequence contexts. **(B)** Circos heat maps of methylation level and gene density distribution along chromosomes of *F. nilgerrensis* in P1 (shoot tips), P2 (callus induction), and P3 (shoot induction). Heatmaps I to V represented the methylation density in the CG, CHG, CHH contexts, TE density, and gene density, respectively.

To comprehensively understand the global DNA methylation dynamics during the tissue culture process of *F. nilgerrensis*, we generated genome-wide methylation profiles of *F. nilgerrensis* for six stages. Global methylation levels showed that CG sites exhibited the highest methylation level in all stages with an average of 49.5%, followed by CHG contexts; CHH contexts were the lowest, with an average of 33.2% and 12.4%, respectively (Supplementary Table S4). These differences could be explained by different types of methylation being regulated by different genes (Cokus et al., 2008). Accordingly, the overall distribution of cytosine methylation levels showed that the CG and CHG contexts had greater proportions of higher methylation levels but relatively smaller changes among different stages compared with CHH contexts (Figure 2A). This result was also supported by the global methylation density distribution map of dedifferentiation and redifferentiation processes (P1–P3), which indicated that the methylation level of CHH contexts obviously changed between different stages and most of these changes occurred in the TE high-density regions (Figure 2B). Consistent with the global methylation patterns, the distribution of methylated cytosines along chromosomes was uneven, of which the proportion of CHH methylation changed dramatically (Supplementary Figure S2).

**FIGURE 3:**
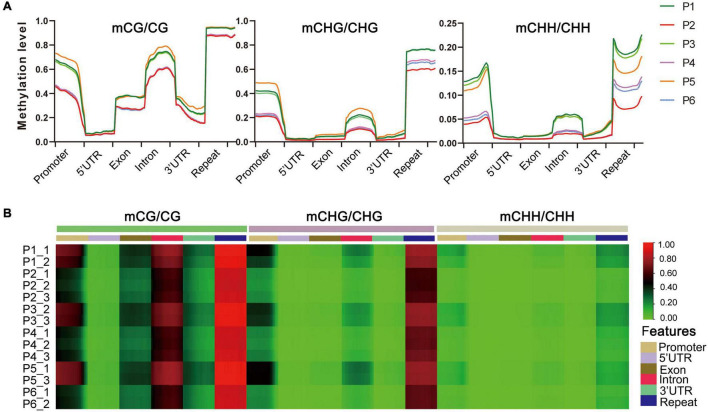
DNA methylation levels in different genomic regions of *F. nilgerrensis*. **(A)** Distribution of DNA methylation among gene component and repeats. **(B)** Heat map of methylation levels of promoter, repeat, coding genes with 5′UTR, exon, intron, and 3′UTR in each sample.

The distribution of DNA methylation levels among genetic regions and repeats was also significantly different, e.g., the DNA methylation levels in all three contexts were much higher in repeats, promoters and introns than in the other regions (5′UTR, exons, 3′UTR) in the six stages (Figure 3A). Heat map analysis produced a similar pattern, which showed different methylation levels in different gene components (Figure 3B). We also found that the methylation levels of P1, P3, and P5 were higher than P2, P4, and P6 stages in each sequence context of genetic regions and repeats, among which P2 (callus induction) exhibited the lowest methylation levels in all three contexts (Figure 3A and Supplementary Table S4). This suggested that the dynamically changed cytosine methylation exhibited in the *in vitro* culture of *F. nilgerrensis* may play an important role in regulating gene expression at different stages, leading to varied phenotypic features.

### Dynamic Pattern of Differentially Methylated Regions Changes Among the Six Stages

**FIGURE 4:**
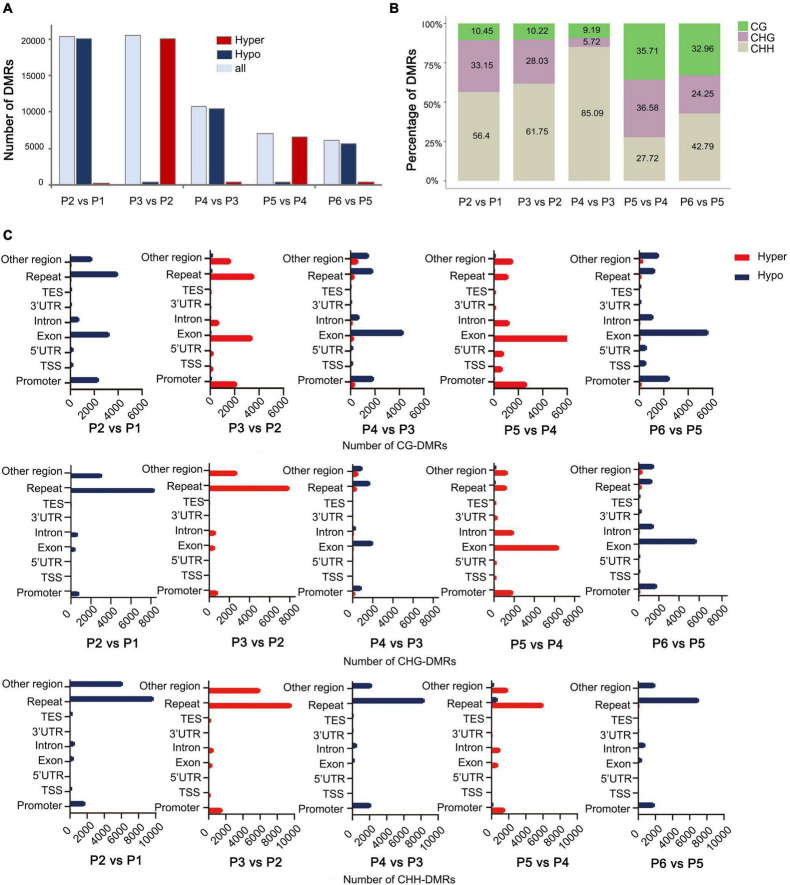
Distribution of DMRs among different stages of tissue culture of *F. nilgerrensis*. **(A)** The number of DMRs in each comparation between adjacent stages. **(B)** Proportion of three contexts (CG, CHG, CHH) in DMRs between each adjacent stage. **(C)** Number of CG/CHG/CHH-DMRs distributed in different genomic regions.

To explore the relationship between DNA methylation and *in vitro* regeneration, we identified DMRs between each adjacent stage. The results showed that many more DMRs were detected in P2 vs. P1 (dedifferentiation) and P3 vs. P2 (redifferentiation) than with other comparisons, suggesting epigenetic regulation plays an important role in reprogramming of gene expression in cell dedifferentiation and redifferentiation. Furthermore, during the tissue culture process, either hypo- or hyper-DMRs alternately dominant (Figure 4A).

Consistent with results from the genome-wide methylation profiles map, the CHH sites, where methylation levels altered dramatically, accounted for a larger proportion of DMRs in the first three comparisons (Figure 4B), but more DMRs were detected in CG and CHG sites in the last two comparisons, including shoot elongation, rooting, as well as outplanting and acclimation (Figure 4B). Further analysis of the CHH methylation distribution on the genetic components indicated that most of them occurred in repeats (most are transposons, TE) and were hypo- and hypermethylated alternatively (Figure 4C) among different stages. Different from CHH-DMRs, a large number of CG-DMRs were distributed in exons and promoters, and a large number of CHG-DMRs occupied either repeats or exons at a different developmental switch (Figure 4C).

### Differentially Methylated Region-Associated Genes and Functional Enrichment Analysis

**FIGURE 5:**
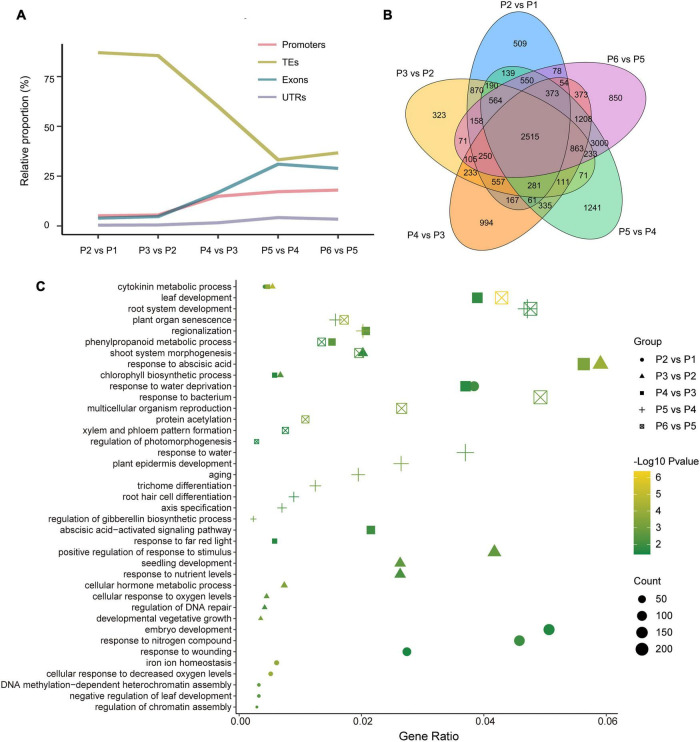
Overview of genome-wide DMR-associated genes (DMGs) during the tissue culture process of *F. nilgerrensis*. **(A)** Dynamic distribution of DMGs in each gene component. **(B)** Venn diagram showing DMGs among the different stage. **(C)** Go enrichment in each comparison between adjacent stages.

Differentially methylated region-associated genes were analyzed based on DMRs that overlapped gene functional regions (such as promoters, UTRs, exons, and introns) with at least 1 bp (Chen et al., 2020). We analyzed the distribution of DMGs components and TEs for the different stages. Interestingly, the changing trend of TE, which account for the highest proportion of DMRs and which decreased dramatically after P3 vs. P2, showed an opposite direction to other genetic components (exons, promoters, and UTRs) (Figure 5A). That was in accordance with the observation mentioned above that TEs were mostly affected during dedifferentiation and redifferentiation at three contexts.

The DMGs detected in each comparison between adjacent stages ranged from 7317 to 11246, and 2515 DMGs were shared in all five comparisons (Figure 5B), suggesting the methylation level of these genes continuously oscillated during different stages of the tissue culture process. To explore the function of these DMGs, GO and KEGG enrichment analyses were conducted. The results indicated that the GO terms involved in hormone metabolic processes, plant development and the response to various environmental factors (including bacteria, far red light, hormone, and hypoxia) were enriched throughout the tissue culture process (Figure 5C and Supplementary Table S5). It was noteworthy that some specific GO terms were also enriched at different stages. DNA methylation-dependent heterochromatin assembly (GO:0006346), embryo development (GO:0009790) and response to wounding (GO:0009611), shoot system morphogenesis (GO:0010016) were enriched in P1–P3, while regionalization (GO:0003002), phenylpropanoid metabolic processes (GO:0009698), leaf and root development (GO:0048366, GO:0022622), as well as multicellular organism reproduction (GO:0032504) were enriched in the P4–P6 stages (Figure 5C).

### Correlation Between DNA Methylation and Expression in a Set of Differentially Methylated Region-Associated Genes

**FIGURE 6:**
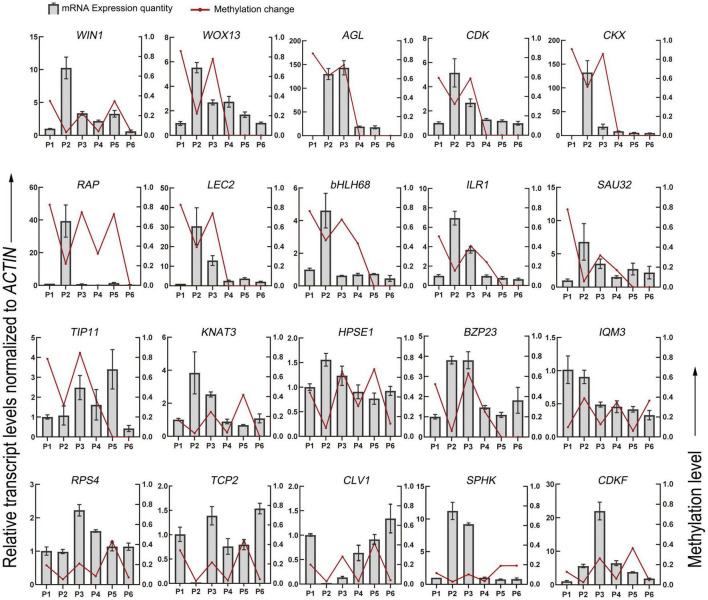
DNA methylation level and gene expression level of DMGs detected in the different stages of tissue culture of *F. nilgerrensis*. The gene expression level was validated by real-time quantitative PCR. The *ACTIN* gene was used as an internal control to standardize the expression of different samples.

Finally, 25 genes, whose methylation levels changed significantly at each stage were listed and expression of 20 genes randomly selected from them were verified by qRT-PCR (Table 2 and Figure 6). Consistent with DNA methylation changes, expression levels of these genes also oscillated during *in vitro* culture, but they only showed relationships with DNA methylation in the first three stages. For example, it is obvious that the most of the promoter hypermethylated genes exhibited reduced expression, including *WIN1*, *WOX13*, *CDK, CKX, RAP, LEC2, bHLH68, ILR1, SAU32, KNAT3*, and *HPSE1*, while three gene bodies (exons and UTRs) hypermethylated genes had an increasing expression trend, including *TCP2, CLV1*, and *CDKF*. No significant expression changes were found in genes after the P4 stage, such as *CDK* and *CKX*, which was consistent with no obvious methylation changes of these genes at these stages. Our findings were roughly consistent with previous reports, that promoter methylation appeared to have a repressive effect on expression, while gene body methylation had a positive effect on expression (Li et al., 2012). Notice that in most of these genes, no correlation was found between DNA methylation and gene expression after the P3 phase, indicating only a partial role of DNA methylation in regulation of gene expression during latter three stages.

**TABLE 2:** List of important genes of methylation differences at different stages of tissue culture of *F. nilgerrensis.*

Stage	Gene name	Properties	Description	Methy. contexts	Diff. methy.	Methy. region
P2 vs. P1 (hypo)	*WIN1*	AP2/ERF transcription factors	Wound inducing protein	CG	–0.32	Promoter
	*WOX13*	Wuschel-related homeobox	Somatic embryogenesis	CG	–0.63	Promoter
	*AGL*	Agamous-like MADS-box protein	Promote the formation of secondary somatic embryos	CG	–0.26	Intron
	*CDK*	Cyclin-dependent kinase	It can promote the formation of callus when it is rich in auxin	CHG	–0.27	Promoter
	*CKX*	Cytokinin dehydrogenase	Cell cycle reentry and progression exhibition	CG	–0.4	Promoter
	*RAP*	Late embryogenesis abundant protein	Late embryonic development protein	CHG	–0.6	Promoter
	*TCP2*	Transcription factor	Eliminate blade characteristics	CG	–0.32	Exon
	*LEC2*	Domain-containing transcription factor	Embryo regaining	CG	–0.43	Promoter
	*bHLH68*	Transcription factor bHLH68	Adjust homeostasis and drought resistance	CHG	–0.3	Promoter
P3 vs. P2 (hyper)	*LEC2*	Domain-containing transcription factor	Embryo regaining	CG	0.45	Promoter
	*KLCR1*	Kinesin light chain-related	During abiotic stress tune	CHG	0.32	Promoter
	*RAP*	Late embryogenesis abundant protein	Late embryonic development protein	CHG	0.53	Promoter
	*CKX*	Cytokinin dehydrogenase	Cell cycle reentry and progression exhibition	CG	0.35	Promoter
	*ILR1*	IAA-amino acid hydrolase ILR1-like 4	Auxin metabolic process	CHG	0.35	Promoter
P4 vs. P3 (hypo)	*SAU32*	Auxin-responsive protein SAUR32	Auxin reactive protein	CG	–0.72	Promoter
	*TIP11*	Aquaporin TIP1-1	Participate in drought stress	CG	–0.47	Promoter
	*CLV1*	Receptor protein kinase CLAVATA1	Maintain the homeostasis of stem cells state	CG	–0.38	Exon
P5 vs. P4 (hyper)	*KNAT3*	Homeobox protein knotted-1-like 3	Heterologous expression promotion somatic embryogenesis	CG	0.38	Promoter
	*GAOX*	Gibberellin 20 oxidase	Overexpression promotes the production of somatic embryos	CG	0.25	Exon
	*CDKF*	Cyclin-dependent kinase	Cell cycle regulator	CG	0.31	Exon
	*RPS4*	RT04_ARATH ribosomal protein S4	Related to resistance to bacterial pathogens	CG	–0.37	Promoter
	*HPSE1*	Heparanase-like protein 1	Binding growth factor and cytokine regulation binding protein white	CHG	–0.55	Promoter
P6 vs. P5 (hypo)	*SPHK*	Sphingosine kinase	Involved in signal transduction in plant cells guide	CG	–0.62	Exon
	*PUB32*	U-box domain-containing protein 32	Involved in ubiquitination and protein qualitative interaction	CG	–0.33	Promoter
	*IQM3*	ARATH IQ domain-containing protein	Young seedlings are closely related to cotyledon expansion	CG	–0.29	Promoter

## Discussion

Epigenetic mechanisms are highly dynamic events that modulate gene expression of plant developmental processes and respond to environmental abiotic stresses (Orowska, 2021). Analysis of the epigenetic landscape of plant tissue culture processes will help to develop methods for reducing or amplifying the mutagenic and epigenetic effects in tissue culture. We herein investigated the genome-wide methylation patterns and differences at the CG, CHG and CHH sites of six developmental stages of tissue culture in *F. nilgerrensis.*

### Global DNA Methylation and Differentially Methylated Regions Detected in Different Stages of Tissue Culture

Among the three contexts, the CG context maintained the highest proportion of total cytosine methylation during the tissue culture process of *F. nilgerrensis*, followed by CHG methylation; the lowest proportion was CHH methylation, mostly distributed in TEs. That is consistent with the previous study in angiosperms that showed CG methylation was the predominant context of DNA methylation, which contributed to more than 50% of total cytosine methylation (Niederhuth et al., 2016). Although CHH methylations accounted for the lowest proportion of total cytosine methylation, they showed the most fluctuating methylation changes among different stages.

Many more DMRs were detected in P2 vs. P1 and in P3 vs. P2, responding to the dedifferentiation and redifferentiation process, respectively, and most of these DMRs were distributed in TEs. That was consistent with previous reports that DNA hypomethylation at the callus stage plays a central role in controlling the activation of the transcription process and the transposition of retrotransposons (Cheng et al., 2006; Fukai et al., 2010). These TE regions were re-methylated in the regenerated plants again for the inhibition of active transposons, which would influence the expression of adjacent genes (Kubis et al., 2003; Zakrzewski et al., 2017). It is noteworthy that the changes of TE proportion and that of other genetic components (exons, promoters and UTRs) in DMRs was opposite after the P3 stage. This indicated that the dedifferentiation and redifferentiation process in the *in vitro* culture systems involved activating or silencing of amounts of transposons, while in other developmental stages, expression of hundreds of genes was epigenetically regulated to control the development of many different cell types.

We found that throughout the tissue culture process of *F. nilgerrensis*, the global pattern of DNA methylation showed dynamic and alternated hypo- and hyper-methylation between each adjacent stage. The dynamics of DNA methylation have already been reported to be an important way to actively reprogram, which plays critical roles in transposon silencing, genome stability and gene expression regulation during cell fate transition in both plants and animals (Feng et al., 2010; Zhang et al., 2010). In plant tissue culture, genome wide hypo- and hypermethylation were predominantly observed during the process of dedifferentiation (callus induction) and redifferentiation (shoots induction), respectively, in a variety of plant species (Neelakandan and Wang, 2012; Ghosh, 2016; Hesami et al., 2020; Lin et al., 2021), that was in accordance with our findings. There is little information concerning alterations in DNA methylation following consecutive stages of tissue culture from explants to outplanting. In *Populus trichocarpa*, the methylomes of explants, calli and regenerated internodes were compared, and the results showed that gene body and transposon 5mC were increased in callus but decreased in regenerated internodes, while promoters 5mC continued to decline among tissues (Vining et al., 2013). Our results showed that methylation levels of all the genetic regions were decreased and increased alternately at the first three stages, roughly corresponding to their three tissues. Furthermore, this trend of alternated hypo- and hypermethylation was continued in the following three stages. It has been reported that different types and concentrations of hormones, together with various stresses and ages of explants would affect the growth and development of culture materials, leading to differences in phenotypes, changing the trend of DNA methylation and induce cell clonal mutations (Law and Jacobsen, 2010; Tiwari et al., 2013). Therefore, the dynamic changes of DNA methylation during *in vitro* culture of *F. nilgerrensis* could be explained by different factors in the microenvironment, such as different types and concentrations of hormones, stages of culture, osmotic stress, light stress, and oxidative stress. In addition, the decreased DMRs in the last three stages indicated that DNA methylation was more stable in the plant tissues with high levels of cell differentiation, suggesting that stage of culture is an important factor affecting DNA methylation levels.

### The Genes Affected by DNA Methylation in Tissue Culture

Many genes with differential DNA methylation were detected at each stage of tissue culture in *F. nilgerrensis*. GO and KEGG analysis of these genes showed that genes involved in hormone metabolic processes, plant development and response to various environmental factors were enriched throughout the tissue culture process. That corresponds to the different stresses in the microenvironment, including different types and concentrations of hormones, osmotic stress, light stress, and oxidative stress. For example, the *IQM3* (IQ domain-containing protein) was involved in plant responses to adversity stress, and the expression of the gene is closely related to seed germination (Zhou et al., 2010); *RPS4* was reported as a member of the TIR-NBS-LRR family, which is involved in resistance to bacterial pathogens (Gassmann et al., 2010).

At each stage there were specific enriched GO terms which contained genes playing a crucial role in adaptation. During callus induction, many genes were hypomethylated in *F. nilgerrensis*, including the key genes *WIN* (Wound-induced protein) and *WOX* (WUSCHEL-related Homeobox) for callus formation. It was reported that *WIN* could induce dedifferentiation and proliferation of cells, and *WOX* could react rapidly to a wound, induce auxin maximization and alter cell fate (Lee and Seo, 2018). During shoot induction, many hypomethylated genes restored methylation, such as *CKX* (Cytokinin dehydrogenase 7) and *ILR1* (IAA-amino acid hydrolase ILR1-like 4), both of which are involved in hormone metabolism. It was reported that cytokinin could promote cell proliferation and shoot induction in the callus (Cortleven et al., 2019), and in *A. thaliana* tissue culture, the absorption of IAA by ILR family mutants is lower than that of the wild type, resulting in shorter hypocotyls and fewer lateral roots (Rampey et al., 2004). Therefore, methylation changes of these genes may reflect crucial roles for regulating the dynamic balance of cytokinin and auxin in *F. nilgerrensis* for shoot induction. For the latter three stages, the key candidate genes with changed methylation were mainly involved in maintaining the steady-state of the stem cell (*CLV1*) (Deyoung et al., 2010; Stahl et al., 2013), regulating the cell cycle (*CDKF*) (Shimotohno et al., 2004; Takatsuka et al., 2009), and participating in ubiquitination and the qualitative interaction of proteins (*PUB32*) (Azevedo et al., 2001; Trujillo, 2018). These genes were closely associated with plant regeneration for stress resistance and development.

It was speculated that DNA methylation affects gene expression by enhancing the binding of certain transcription activators or inhibiting the binding of certain transcription repressors (Zhang et al., 2018). Based on the results of qRT-PCR, we found that they did not exhibit consistent relationships between different genetic regions and different stages. In the first three stages, a negative correlation between DNA methylation and gene expression was found in the promoter, while it seemed that a positive correlation exist in the gene bodies. However, no correlation was detected between methylation and gene expression in the latter three stages. Obviously, several other genetic and epigenetic factors should also be involved in regulating shoot elongation, rooting and outplanting in *F. nilgerrensis*. Our results suggest that the widely accepted belief that hypermethylation leads to repression and hypomethylation leads to activation of genes is an oversimplification, and that this generalization is applicable only in a small fraction of DMGs (Dafni et al., 2018).

In conclusion, we accurately monitored the methylation patterns of consecutive steps of tissue culture by measuring the whole genome DNA methylation levels of *F. nilgerrensis*. We found that the majority of DMRs were located in the TE high-density regions in the dedifferentiation and redifferentiation stages, whereas the proportion of gene-body DMRs gradually increased in the later stages of tissue culture. In addition, we also obtained a series of candidate genes which are closely associated with plant regeneration. This information gives a deeper insight into the relevance of DNA methylation and somatic clonal variation, which can be used to facilitate molecular breeding.

## Data Availability Statement

The data presented in the study are deposited in the NCBI Sequence Read Archive (BioProject PRJNA778971), accession numbers SRR16914072, SRR16914071, SRR16914070, SRR16914069, SRR16914068, and SRR16914067.

## Author Contributions

TZ and QQ conceived and designed the study. QC, YF, XD, LH, and JL performed the experiments and analyzed the data. QC, YF, PT, MC, TZ, and QQ analyzed the data, wrote the manuscript, and exercised general supervision. All authors read and approved the final manuscript.

## Conflict of Interest

The authors declare that the research was conducted in the absence of any commercial or financial relationships that could be construed as a potential conflict of interest.

## Publisher’s Note

All claims expressed in this article are solely those of the authors and do not necessarily represent those of their affiliated organizations, or those of the publisher, the editors and the reviewers. Any product that may be evaluated in this article, or claim that may be made by its manufacturer, is not guaranteed or endorsed by the publisher.
