# Supplementary material for: Dynamic Changes of DNA Methylation During Wild Strawberry (Fragaria nilgerrensis) Tissue Culture
Source: Front Plant Sci. 2021 Nov 30;12:765383. doi: 10.3389/fpls.2021.765383 (PMC8669611; doi:10.3389/fpls.2021.765383)
Supplement: Supplementary file 2 [file Data_Sheet_2.PDF]

Dynamic changes of DNA methylation during wild strawberry (*Fragaria nilgerrensis*) tissue culture

Qiang Cao[1†], Yuxi Feng[1†], Xiongwei Dai[1†], Lin Huang[1], Jiamin Li[1], Pang Tao[2], M. James C. Crabbe[3, 4, 5], Ticao Zhang[6*], Qin Qiao[1*]

[†]These authors have contributed equally to this work and share first authorship.

[1]School of Agriculture, Yunnan University, Kunming, China

[2]Horticultural Research Institute, Yunnan Academy of Agricultural Sciences, Kunming, China

[3]Wolfson College, Oxford University, Oxford, UK

[4]Institute of Biomedical and Environmental Science & Technology, School of Life Sciences, University of Bedfordshire, Park Square, Luton, UK

[5]School of Life Sciences, Shanxi University, Taiyuan, China

[6]College of Chinese Material Medica, Yunnan University of Chinese Medicine, Kunming, China

*** Correspondence:**
Ticao Zhang
ticaozhang@126.com
Qin Qiao
qiaoqin@ynu.edu.cn

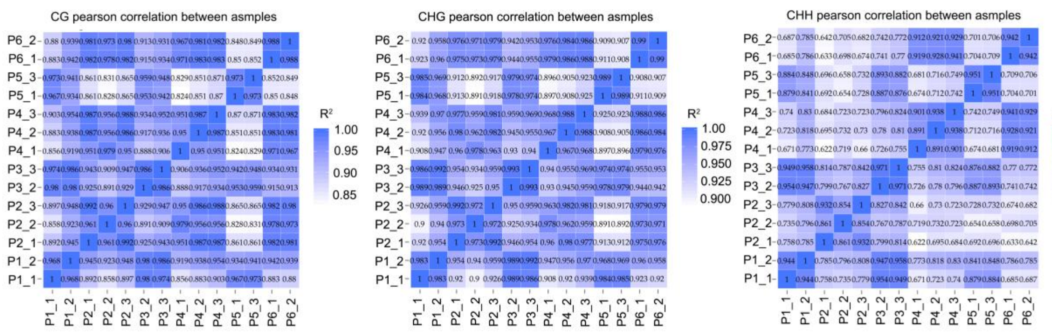

Figure S1. Sample correlation analysis.

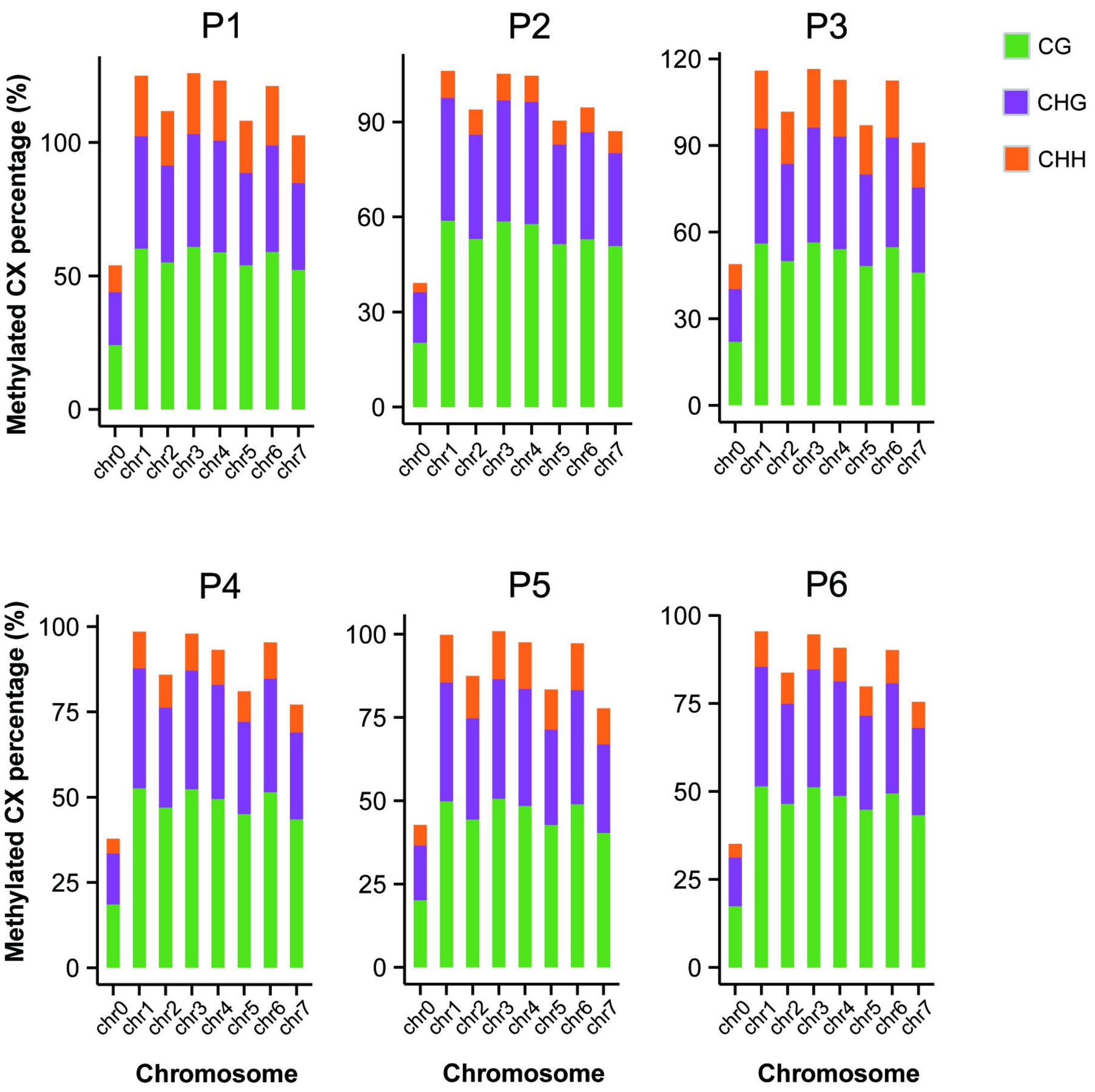

Figure S2. Chromosome distribution of global DNA methylation patterns in each sample in the context of CG, CHG, and CHH. Chr0-Chr7 on the x-axis represent chromosomes 0-7 of *F. nilgerrensis*, (chr0 is the contig fragments that are not mounted to the chromosome).
